# The Association between Sleep Duration and Metabolic Syndrome: The NHANES 2013/2014

**DOI:** 10.3390/nu11112582

**Published:** 2019-10-26

**Authors:** Abbas Smiley, David King, Aurelian Bidulescu

**Affiliations:** 1 Department of Surgery, Westchester Medical Center, School of Medicine, New York Medical College, New York, NY 10595, USA; 2 Henry M Jackson Foundation for the advancement of Military Medicine, Bethesda, MD 20817, USA; drdavidbking@gmail.com; 3 Department of Epidemiology, School of Public Health, Indiana University, Bloomington, IN 47404, USA; abidules@indiana.edu

**Keywords:** sleep, metabolic syndrome, metabolic syndrome severity score, generalized additive model, effect modification

## Abstract

Background: We aimed to assess the association of sleep with metabolic syndrome in the 2013/2014 National Health and Nutrition Examination Survey (NHANES). Methods: Sample size included 2737 out of 2013 and 2014 NHANES surveys. Cross-sectional study of metabolic syndrome and sleep duration was conducted. Metabolic syndrome was defined according to NCEP ATPIII (National Cholesterol Education Program Adult Treatment Panel III) criteria. Metabolic syndrome severity score was calculated based on actual measurement of each component, adjusted for sex and race. The generalized additive model (GAM) was built to assess the smooth relationship between metabolic syndrome/metabolic syndrome severity score and sleep duration. Adjustment of models were done for age, sex, race, and sitting time. The value of effective degree of freedom (EDF) formed by the GAM model shows the degree of curvature of the relationship. A value of 1 for EDF is translated as the linear shape of relationship. Values larger than one denote a more complex relationship between the response variable and the predicting one. Results: There was a U-shaped association between sleep duration and metabolic syndrome in univariable GAM (EDF = 2.43, *p* = 0.06) and multivariable GAM (EDF = 2.03, *p* = 0.20). The lowest risk of metabolic syndrome was observed in people sleeping 7 hours/night. There was a significant U-shaped association between sleep duration and metabolic syndrome severity score in multivariable GAM (EDF = 2.94, *p* = 0.0004). Similarly, the lowest mean metabolic syndrome severity score was observed in people sleeping 7 hours/night. There was an effect modification of sex and sleep duration indicating strong U-shaped relationship of metabolic syndrome severity score and sleep duration in women (EDF = 3.43, *p* = 0.00002) and semi-linear association in men (EDF = 1.76, *p* = 0.04). Conclusion: Short and long sleep duration was associated with higher risk of metabolic syndrome and higher scores of metabolic syndrome severity score in women. Short sleep duration was associated with higher risk of metabolic syndrome and higher scores of metabolic syndrome severity score in men.

## 1. Introduction

The recommend daily duration of sleep for adults is 7–8 hours [1]. Sleeping <7 hours could be detrimental for overall well-being, health, and performance [2]. Literature shows some associations between metabolic syndrome and sleep duration [3,4,5,6,7,8]. The associations were not consistent for both genders. The association of short and/or long sleep duration was also insignificant in some other studies [9,10,11,12,13]. Overall, no universal agreements were found on the significance of associations for both short and long sleep durations. Moreover, the long duration of sleep was defined differently in various studies, more than 7, 8, 9, or 10 hours. The inconsistencies justified further studies along with the application of more sophisticated methods of analysis to draw the non-linear associations of metabolic syndrome and sleep. 

## 2. Methods

The current study was conducted to assess the association of sleep duration and metabolic syndrome prevalence in the National Health and Nutrition Examination Survey (NHANES) 2013/2014. 

### 2.1. Population

National Center for Health Statistics is run by the Centers for Disease Control and Prevention (CDC). NHANES began about 60 years ago and aimed to assess health and nutritional status of people in the United States. NHANES focused on various populations and different health topics. The prevalence of major diseases and their risk factors were measured in order to evaluate the relationship of nutritional status and health promotion, to advance national biological standards, and to plan new health programs and services. The surveys in NHANES covered a sample size of 5000 per year who were representative of the US nation. They were selected from various counties across the US, 15 of which are visited each year. The surveys are unique because they include both interviews and physical examinations. The latter involves clinical, physiological, and laboratory evaluations managed by trained medical personnel. The current study covered 10,175 interviews and physical examinations. The dataset included demographic, socioeconomic, dietary, and health-related information. Information about sleep was gathered in 6464 people of which, components of metabolic syndrome were recorded in 2840 people. Metabolic syndrome was defined when 3 or more of the following findings were present according to NCEP ATPIII criteria: [14] central obesity, high fasting glucose, high serum triglyceride, high serum high density lipoprotein (HDL) cholesterol, and high blood pressure. Metabolic syndrome severity score was calculated according to the formulae presented by Lee, Gurka, and DeBoer [15].

### 2.2. Data Analysis

The data were examined and outliers that were out of range of mean ± 2SD (standard deviation) for sleep duration, were deleted. Due to the low number of the sample size and in order to have better smoothing function in GAM, people sleeping 10 hours were regrouped into the 9-hour sleeping category. The descriptive characteristics for each variable were analyzed. Presence of metabolic syndrome was treated as categorical outcome. Metabolic syndrome severity score was calculated and treated as continuous outcome.

Assuming the U-shape association cardio-metabolic outcomes and sleep duration in the literature, a penalized smoothing spline was employed [16]. Non-linear relationship of sleep with metabolic syndrome/metabolic syndrome severity score was assessed through generalized additive models (GAM). In our analysis, we fit 5 GAM models to NHANES data. The first three models used metabolic syndrome (yes/no) as a binary response whereas the fourth and fifth models used metabolic syndrome severity score as the response which is continuous.

ResponsePredictorModel 1: Metabolic Syndrome (Yes/No)Smooth(Sleep)Model 2: Metabolic Syndrome (Yes/No)Age, Sex, Race, Sitting, smooth(Sleep)Model 3: Metabolic Syndrome (Yes/No)Age, Race, Sitting, smooth(Sleep*Sex)Model 4: Metabolic Syndrome Severity ScoreAge, Sex, Race, Sitting, smooth(Sleep)Model 5: Metabolic Syndrome Severity ScoreAge, Race, Sitting, smooth(Sleep*Sex)

Model 1 used metabolic syndrome (yes/no) as a binary response and a smoothing spline function of sleep as a univariable predictor. Model 2 was a multivariable version of model 1; it used metabolic syndrome (yes/no) as a binary response but the predictor variables were age, sex, race, sitting time, smooth(sleep). Model 3 was similar to model 2 but the interaction between sex and the smoothing function of sleep was added to the model. Model 4 was similar to model 2 but used the continuous response variable of metabolic syndrome severity score as the response variable; it used age, sex, race, sitting time, smooth(sleep) as predictor functions. Model 5 was similar to model 4 but the interaction between sex and the smoothing function of sleep was added to the model. 

GAM is an extension of the generalized linear model which allows the evaluation for the curvilinear relationship of the outcome and the predictors. Model assumptions where assessed by investigating the normality of residuals, homoscedasticity, and residual symmetry. Age, sex, race, and sitting time were applied to adjust GAM models. Since waist circumference and body mass index (BMI) showed very strong correlation (r = 0.91, *p* < 0.0001) and waist circumference is part of the dependent variable (metabolic syndrome), it was not appropriate to consider BMI among confounders and further adjust the model for BMI. If the model was adjusted for BMI, it would remove any association existed between waist circumference and other independent variables (age, sex, race, sitting time, and sleep duration). 

The reported values of effective degree of freedom (EDF) output demonstrate the degree of curvature of the smooth. Value of 1 is the sign of linear pattern of relationship. Value of EDF >1 is the sign of a more complex relationship between metabolic syndrome and sleep duration. The basic residual plots were checked to assure good compliance with model assumptions. The predicted smooth functions along with the confidence intervals were plotted in univariable and multivariable GAM models. A *p*-value less 0.05 was considered significant. Statistical analyses were carried out using R-3.4.3 [17].

## 3. Results

Our sample contained 2737 people, with the age range of 18–80 years. The mean (SD) age was 47.7 (18.3) years and 52.3% were women. A total of 61% of the sample were married, 44% had household income >$45,000/year, and 53% were educated in college or above. The mean (SD) duration of sleep was 6.82 (1.22). Characteristics of study subjects according to metabolic syndrome are presented in Table 1. About 31.5% had metabolic syndrome. Subjects with metabolic syndrome were significantly younger than those without metabolic syndrome. Also, they were significantly different in terms of age, ethnicity, marital status, education, smoking, household size, depression, diastolic and systolic blood pressure, HDL-cholesterol, fasting blood glucose, triglyceride, and waist circumference (Table 1). White and Asian races had the highest and the lowest prevalence of metabolic syndrome compared to other ethnicities. Women had higher prevalence of metabolic syndrome than men. Low educated people also had higher prevalent metabolic syndrome. 

**Figure 1:**
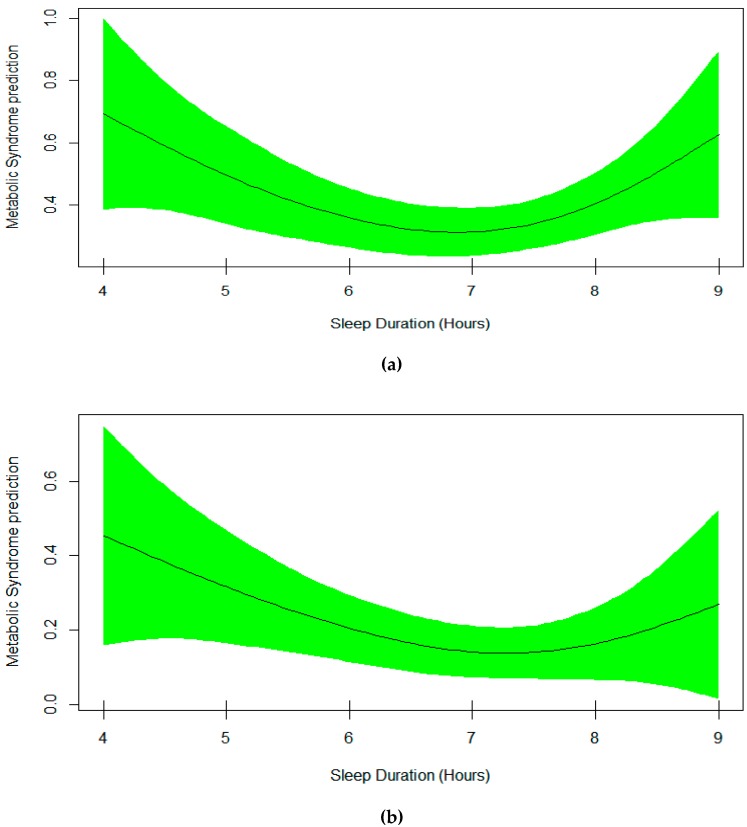
Plots of estimated smoothing spline function of sleep duration with 95% confidence band for the generalized additive model when the response variable was metabolic syndrome. (**a**) Model 1 shows the univariable smooth function of sleep duration (EDF = 2.428, *p* = 0.06). (**b**) Model 2 represents the multivariable smooth function of sleep duration (EDF = 2.03, *p* = 0.20).

**Figure 2:**
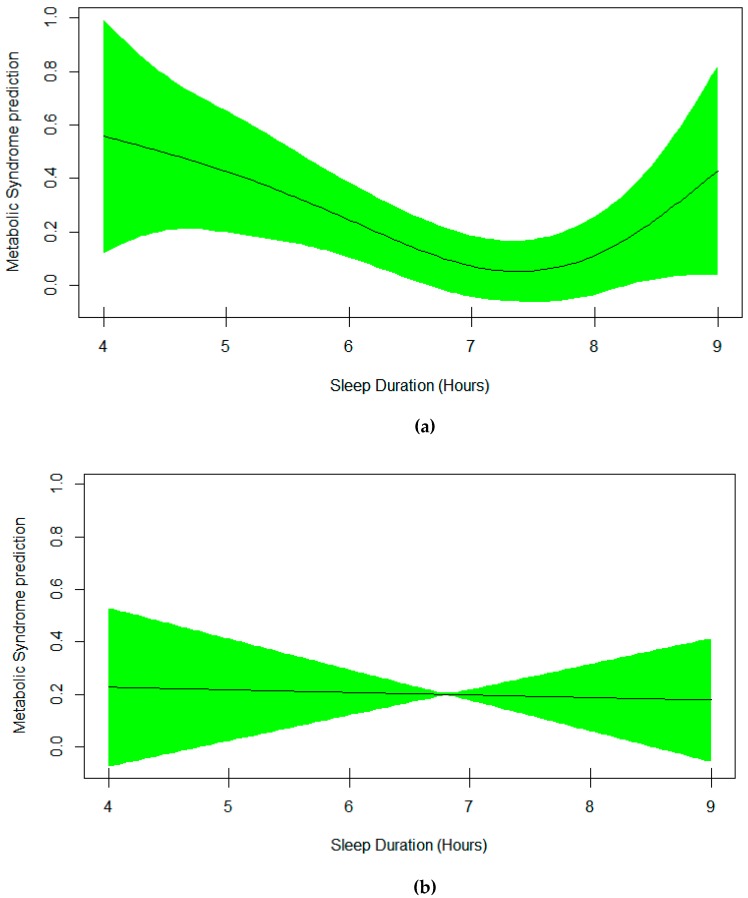
Effect modification of sex and sleep duration in the multivariable generalized additive model 3. (**a**) EDF was 2.57 (*p* = 0.06) in female model (upper plot), (**b**) EDF was 1.002 (*p* = 0.80) in male model (lower plot).

Model 1 which used metabolic syndrome (yes/no) as a binary response, and a smoothing spline function of sleep as a univariable predictor is shown in the upper plot in Figure 1. Model 2, which was a multivariable version of model 1 but further adjusted for additional variables, is shown in Table 2 and the lower plot in Figure 1. Technically, Table 2 presents the output of the multivariable generalized additive model for metabolic syndrome. It includes the smoothing estimate for sleep duration in association with metabolic syndrome adjusted for age, sex, and sitting time. An EDF of 2.03 indicates a non-linear fit between sleep duration and metabolic syndrome. Plot of predicted smooth association of metabolic syndrome and sleep duration, which also includes 95% confidence intervals, is shown in Figure 1. The plot shows a U-shape association of sleep duration and the risk of metabolic syndrome in both univariable and multivariable GAM models. The lowest risk is observed in those sleeping 7–7.5 hours per night. Furthermore, evaluation of effect modification of sex and sleep duration by using multivariable GAM model 3 revealed 2 different smoothing shapes; U-shaped in women and linear in men (Figure 2).

**Figure 3:**
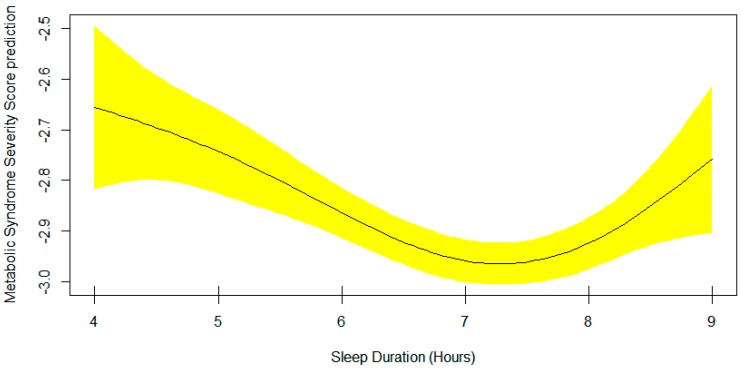
Plots of estimated smoothing spline function of sleep duration with 95% confidence band for the multivariable generalized additive model 4 when the response variable was metabolic syndrome severity score (EDF = 2.94, *p* = 0.0004).

**Figure 4:**
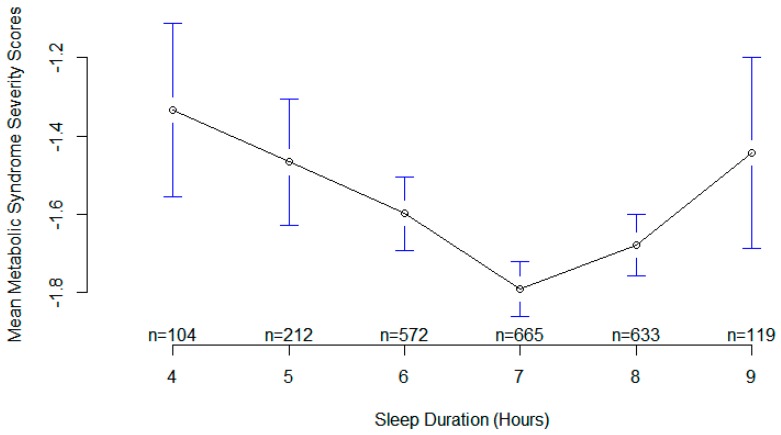
Plots of sleep duration vs. mean metabolic syndrome severity score.

Table 3 presents the output of the multivariable generalized additive model 4 for the metabolic syndrome severity score. It includes the smoothing estimate for sleep duration in association with metabolic syndrome severity score adjusted for age, sex, race, and sitting time. A significant EDF of 2.94 indicates a non-linear fit between sleep duration and metabolic syndrome severity score. The plot of predicted smooth association of metabolic syndrome severity score and sleep duration which includes 95% confidence intervals is shown in Figure 3. The plot shows a significant U-shape association of sleep duration and the metabolic syndrome severity score. The lowest mean score is observed in those sleeping 7–7.5 hours per night. Also, the mean plot of metabolic syndrome severity score according to sleep duration is demonstrated in Figure 4. Interestingly, the sample size for every specific sleep duration is demonstrated on *X*-axis. The lowest mean metabolic syndrome severity score is observed in those sleeping 7 hours/day (Figure 4). Remarkably, people sleeping 5 hours or 9 hours show almost similar mean metabolic syndrome severity scores (Figure 4). 

**Figure 5:**
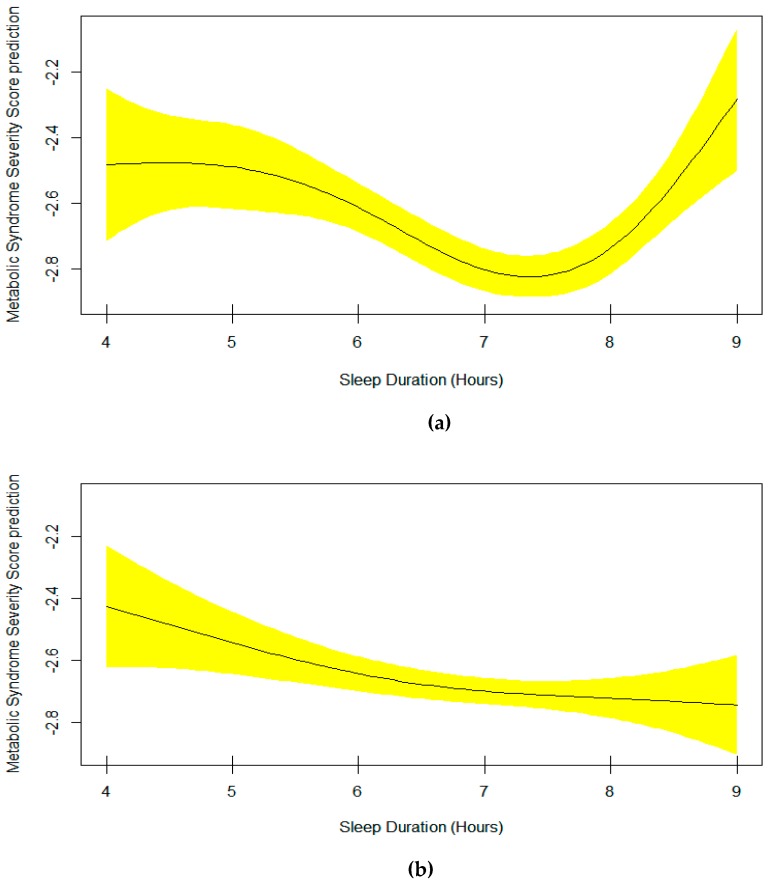
Effect modification of sex and sleep duration in the multivariable generalized additive model 5 when the response variable was the metabolic syndrome severity score. (**a**) EDF was 3.43 (*p* = 0.00002) in the female model (upper plot), (**b**) EDF was 1.76 (*p* = 0.04) in the male model (lower plot).

**Figure 6:**
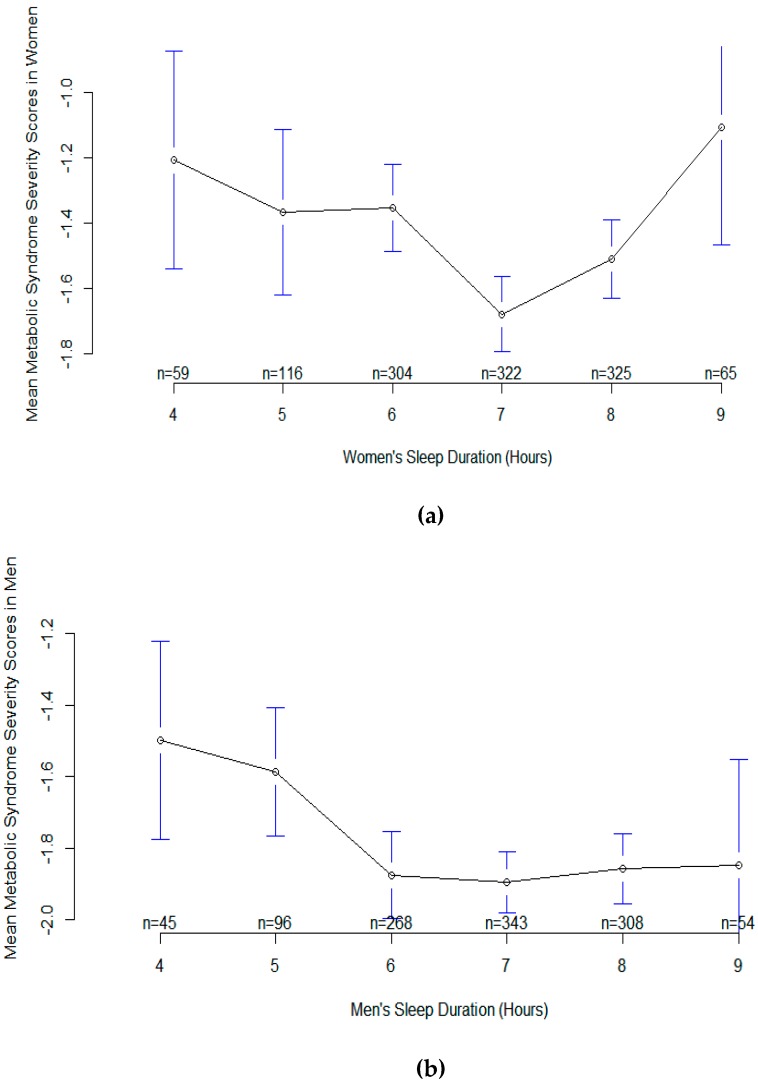
Plots of sleep duration vs. mean metabolic syndrome severity score in women (upper plot) and men (lower plot). (**a**) Plot of sleep duration vs. mean metabolic syndrome severity score in women. (**b**) Plot of sleep duration vs. mean metabolic syndrome severity score in men.

Furthermore, evaluation of effect modification of sex and sleep duration by using the multivariable GAM model 5 showed two different smoothing patterns for metabolic syndrome severity score; U-shaped in women (EDF = 3.43, *p* = 0.00002) and semi-linear in men (EDF = 1.76, *p* = 0.04) (Figure 5). The association was significant for both genders. Short sleep duration showed similar relationships with metabolic syndrome severity score in men and women. But the pattern of long sleep duration was different; sharply increased metabolic syndrome severity score in women and no change or slightly decreased metabolic syndrome severity score in men (Figure 5). Also, the mean plot of metabolic syndrome severity score according to sleep duration is demonstrated in Figure 6. Again, the sample size for every specific sleep duration is demonstrated on *X*-axis. The lowest mean metabolic syndrome severity scores for both men and women were observed in people sleeping 7 hours per day (Figure 6) although the metabolic syndrome severity scores for sleep durations of 6, 7, 8, and 9 hours in men were more or less similar.

## 4. Discussion

Few studies evaluated the relationship of sleep duration and cardiometabolic outcomes in the NHANES database. We evaluated the cross-sectional association of sleep duration and metabolic syndrome/metabolic syndrome severity score through the generalized additive model. In both univariable and multivariable metabolic syndrome/metabolic syndrome severity score models, EDF was greater than 2, indicating the curved association of sleep and metabolic syndrome/metabolic syndrome severity score. This means assuming linearity for the association of sleep duration with metabolic syndrome/metabolic syndrome severity score is not appropriate. The lowest risk of metabolic syndrome was observed in people sleeping 7 hours per night. Similarly, the mean score of those sleeping less than 7 hours or more than 7 hours was higher than that in those sleeping 7 hours. We may be able to predict the risk of metabolic syndrome or the score of metabolic syndrome severity score through the final models having age, sex, race, sitting time, and sleep duration. Short sleep duration had similar association with risk of metabolic syndrome in men and women. Nevertheless, models with effect modification of sex showed remarkably stronger association of long sleep duration and metabolic syndrome severity score in women vs. men. The possible mechanisms of association of sleep and metabolic syndrome have been discussed elsewhere. In summary, stage 3 is the most important stage of sleep since the growth hormone (GH) and GH releasing hormone (GHRH) are released at this time. They induce fat burning, bone building, and general repair and regeneration. The longest part of stage 3 in sleep takes place before midnight. Delayed sleep onset until midnight or later, would suppress the largest GH pulse. Sleep restriction induces high levels of ghrelin and low levels of leptin. Ghrelin stimulates appetite whereas leptin does the reverse. Advanced glycation end products (AGEs) are significantly increased in chronic sleep insufficiency and are also associated with insulin resistance in males with chronic sleep insufficiency. Sleep insufficiency increases sympathetic activity and pro-inflammatory cytokines, both of which increase insulin resistance. Accumulations of extracellular *β* amyloid protein plaques and intracellular tau neurofibrillary tangles in brain tissues start immediately after one night of sleep insufficiency. These plaques and tangles are neurotoxins that potentiate each other’s destructive effects on the structures and functions of brain cells and cause neuronal death. The consequence is a global decrease in cognition and decision making, manifested in increased consumption of fatty foods and unhealthy snacks in late sleepers. High levels of β amyloid and proteins might lead to sleep fragmentation, worsening of sleep quality, and daytime somnolence. Concentration will be more difficult, and performance will be reduced [18].

The main strengths of the current study were the method of analysis and the employment of the metabolic syndrome severity score. Application of the generalized additive model to explore the nonlinear association of sleep and metabolic syndrome/metabolic syndrome severity score improved the risk adjustment compared to linear models or categorization of linear terms [19,20]. Categorizing the sleep duration, using dummy variables on categories for adjusting the risk and using linear/logistic regression for nonlinear associations may induce some residual confounding [21,22,23,24,25,26]. In addition, calculating the metabolic syndrome severity score improved the strength of association because first, it provided a continuous measure of risk of metabolic status whereas metabolic syndrome is just a categorical measure of yes or no; second, the metabolic syndrome severity score is sex and race specific whereas in metabolic syndrome only HDL-cholesterol and waist circumference are sex specific; third, all five components of metabolic syndrome actually contribute in the score calculation, whether they are high, borderline, or low, whereas in metabolic syndrome, only the high components defined based on one-point threshold are considered to diagnose metabolic syndrome [27]. Imagine a person with three borderline components and two high components vs. a person with three high components and two normal components. The metabolic condition of the first person could be worse than the metabolic condition of the second one. But according to the definition of metabolic syndrome, only the second one would be diagnosed with metabolic syndrome, not the first one. This shortcoming would be tackled by calculating the metabolic syndrome severity score which includes the actual measurements of all five components. The precision of the metabolic syndrome severity score in predicting the risk of health outcomes has been demonstrated by other studies [28,29,30,31,32,33]. Interestingly, investigation on the components of metabolic syndrome in NHANES 2013/2014 demonstrated significant U-shape association of sleep duration and triglyceride levels and reverse U-shape association of sleep duration and HDL cholesterol [34]. Similar findings on the association of sleep duration and metabolic syndrome/metabolic syndrome severity score/metabolic syndrome components were observed in two other datasets, the Reasons for Geographic and Racial Differences in Stroke (REGARDS) and the Jackson Heart Study [35].

The limitations of the current study are as follows: Its cross-sectional design prohibits inferring the causal association metabolic syndrome and sleep duration. Quality of sleep such as difficulty initiating or maintaining sleep, regularity/irregularity of sleep-wake schedules, and excessive daytime sleepiness 14 were not regularly assessed in NHANES surveys. Seasonal variations in duration of sleep and recall bias might induce information bias. BMI could have been potentially considered as an additional confounder in the final model in our study because it is associated with both metabolic syndrome and sleep duration. But, as mentioned above, BMI showed very strong correlation with waist circumference (r = 0.91, *p* < 0.0001) and since waist circumference is part of the definition of metabolic syndrome, the final model was not further adjusted for BMI. Then, it was not possible to separate the relationship of central obesity and sleep duration vs. the relationship of general obesity (i.e., BMI) and sleep duration. Finally, having the detail of dietary intake related to metabolic syndrome may improve the evaluation of metabolic syndrome and sleep association.

Given the current prevalent lack of enough sleep and the growing prevalence and incidence of metabolic syndrome and obesity, finding the U-shaped relationship of sleep duration and metabolic syndrome may target sleep as a serious risk factor for cardiovascular outcomes. Longitudinal studies may improve the reliability and the generalizability of findings.

## Figures and Tables

**Table 1:** Characteristics of participants in the National Health and Nutrition Examination Survey (NHANES) 2013/2014 according to metabolic syndrome.

Participants’ Characteristics		Metabolic Syndrome		
		No, *n* = 1735	Yes, *n* = 793	*p*
Age, Years, Mean (SD)		48.5 (18.6)	46.5 (17.6)	0.02
Sex, *n* (%)	Male	836 (33.1%)	376 (14.9%)	0.0001
	Female	899 (35.6%)	417 (16.5%)	
Ethnicity, *n* (%)	Hispanic	364 (14.4%)	186 (6.9%)	0.0001
	White	729 (28.8%)	388 (15.3%)	
	Black	345 (13.6%)	142 (5.6%)	
	Asian	250 (9.9%)	55 (2.2%)	
	Multiracial	47 (1.9%)	16 (0.6%)	
Marital Status, *n* (%)	Married or Living with partner	972 (40.7%)	495 (20.7%)	0.0001
	Widowed	104 (4.3%)	69 (2.9%)	
	Divorced or Separated	191 (8.0%)	130 (5.4%)	
	Never married	345 (14.4%)	85 (3.6%)	
Education, *n* (%)	<9th grade	104 (4.1%)	79 (3.1%)	0.0001
	9–11th grade	201 (8.0%)	138 (5.5%)	
	High school	334 (13.2%)	173 (6.8%)	
	Some college	481 (19.0%)	247 (9.8%)	
	≥College graduate	490 (19.4%)	141 (5.6%)	
Sitting, Minutes/Day, Mean (SD)		434 (527)	470 (709)	0.1
Sleep Duration, Hours, Mean (SD)		6.82 (1.2)	6.80 (1.3)	0.6
Diastolic Blood Pressure, mmHg, Mean (SD)		67.5 (11.2)	71.5 (14.5)	0.0001
Systolic Blood Pressure, mmHg, Mean (SD)		117.5 (15.7)	130.5 (17.8)	0.0001
Fasting Blood Glucose, mg/dL, Mean (SD)		98.2 (21)	123.5 (46)	0.0001
High Density Lipoprotein, mg/dL, Mean (SD)		58.5 (15)	44.0 (13)	0.0001
Triglyceride, mg/dL, Mean (SD)		93.2 (52)	193.7 (129)	0.0001
Waist Circumference, cm, Mean (SD)		92.5 (14.4)	110.1 (14.9)	0.0001

**Table 2:** Association of metabolic syndrome and independent variables measured by the multivariable generalized additive model 2.

R^2^ = 0.064 *n* = 2527	Outcome: Metabolic Syndrome	
	*B*	*p*
Age	0.03	0.0001
Sex		
Male	Reference Group	
Female	0.04	0.60
Race		
Hispanic	Reference Group	
White	−0.12	0.30
Black	−0.32	0.01
Asian	−0.88	0.0001
Multiracial	−0.25	0.40
Sitting	0.00007	0.30
Sleep	Smooth Curve, EDF = 2.03	0.20

**Table 3:** Association of metabolic syndrome severity score and independent variables measured by the multivariable generalized additive model 3.

R^2^ = 0.19 *n* = 2305	Outcome: Metabolic Syndrome	
	*B*	*p*
Age	0.013	0.0001
Sex		
Male	Reference Group	
Female	0.38	0.0001
Race		
Hispanic	Reference Group	
White	0.35	0.0001
Black	0.95	0.0001
Asian	0.09	0.20
Multiracial	0.27	0.04
Sitting	0.00009	0.004
Sleep	Smooth Curve, EDF = 2.94	0.0004
